# A Remarkable Case of Paralysis of the Lower Extremities, Successfully Treated by an Issue

**Published:** 1782

**Authors:** 


III. *A remarkable caſe of Paralyſis of the Lower Extremities, ſucceſsfully treated by an Iſſue. Communicated in a letter to* Samuel Foart Simmons, M. D. F. R. S. *by Mr.* Charles Kite, *Surgeon at Graveſend in Kent. Read* Dec. 2, 1782.

A Lad, in the twelfth year of his age, of a fair complexion and a ſlender make, was, in Sept. 1780, ſeized with a cold ſhivering, which continued about five minutes. A fever ſucceeded and ran pretty high the whole night, but but towards morning abated. A contraction of his legs enſued, attended with ſo much debility that he was unable to walk. He complained of pains all over his body, but more particularly about his ſtomach and the ſmall of his back. Theſe ſymptoms induced me to treat it as a fever, ariſing from cold, and irregularity. An emetic and ſome antimonial medicines were preſcribed, by which he was in ſome degree relieved.

The fever ſometimes remitted, and at other times a complete intermiſſion took place, but without obſerving any regular period. When the paroxyſm came on, I was never able to diſcover, that it attacked with ſhivering or even chillneſs. The power of uſing his legs daily diminiſhed, and about the tenth day he was utterly incapable of moving them in the leaſt off the ground.

It occurred to me, that this paralyſis might probably be the conſequence of ſome tumour on the ſpine. I examined his back carefully, but at this time no ſwelling was perceptible. The cauſe, therefore, appeared to me to be hid in impenetrable darkneſs. I attempted the uſe of ſeveral medicines; but the boy poſitively refuſed to take any of them. All I did, therefore, was to have have the thighs and legs well rubbed with a ſti-mulating embrocation; to apply a bliſter to the calf of each leg, and one to the ſacrum, where the nerves to the lower extremities paſs out. This plan, on which my whole dependance was placed, did not by any means anſwer my expec-tation: but on the contrary, every ſymptom was manifeſtly aggravated, with the addition of many circumſtances which ſeemed to predict a ſpeedy and fatal termination. I called on him at the end of a month from the time of the at-tack. He appeared then in a more miſerable ſituation; had a ſhort cough, a quick and weak pulſe, a purging, very little appetite, and the *facies hippocratica* to the greateſt degree I ever obſerved. It was with the utmoſt difficulty he could ſit in his chair a few minutes; and when he did, the muſcles of his back were ſo re-markably weak they could not ſupport his body, but were bent double. It was extremely diſ-treſſing to him to be moved in bed; and when any one either bent or extended his legs, it was attended with great pain, particularly in the hips.

In the courſe of my inquiries, I was informed a ſwelling had within three or four days appeared on the back. On examination, I found it to be of the ſize of a large duck's egg, but neither much much inflamed nor very painful, comprehending the third and fourth vertebræ lumborum. The ſpine was not disfigured; nor could I perceive the ſpinal proceſſes any ways affected. Its appearance might very properly be compared to a large abſceſs beginning to form.

The boy now obſerved, he always had felt an uneaſineſs in that part ever ſince he received a blow there with a ſtone, which, he ſaid, happened in the morning of the day he was taken ill; and he recollected that it was with difficulty he could walk home, (which was about a mile diſtant) on account of a pain and weakneſs in his back. This was the firſt time he had ever mentioned the circumſtance of the blow. I was now perfectly convinced that all his ſymptoms proceeded from the injury done to the ſpinal marrow, or its membranes, by this accident; and immediately recollected the advice given us in ſimilar caſes by that eminent ſurgeon, Mr. Pott. But his danger ſeemed ſo great, and the probability of any thing giving him relief ſo little, that it was with a view to avoid the imputation of inhumanity, and to neglect nothing that ſeemed to promiſe even a probability of relief, rather than with any expectation of ſucceſs, that I determined to try the effect of an iſſue.

As he lived in the country, and there was no time to loſe, inſtead of applying a cauſtic, I immediately made an inciſion the whole length of the tumour, thro' the ſkin and adipoſe membrane down to the *faſcia lumborum*. Into this wound I put two ſmall beans. The parts did not appear much diſeaſed; no matter or any other fluid was evacuated, and the faſcia was uninjured, and of a ſilver hue.

The patient continued in nearly the ſame ſtate as before for three or four days, about which time the wound ſuppurated favourably. In the ſhort ſpace of a week the good effect of this diſcharge became evident; his countenance was more enlivened, his pulſe not ſo quick, and he could move his legs a little in bed. From this period he mended amazingly. At the end of a fortnight his appetite was good; his fever had entirely left him in the day, and he was but little diſturbed by it in the evening.

In three weeks, with a trifling aſſiſtance, he was capable of walking round the room. All this time the iſſue diſcharged freely; and in proportion as it diſcharged, the ſwelling gradually diminiſhed; ſo that in about one month from the time the inciſion was made, no remains of the tumour could be perceived.

 His

His health every day improved confiderably, and in five weeks he was fo perfectly recovered, that his mother concluding there could be no further occafion for the iffue, fuffered it to heal.

For the firft three months he walked a little limping, and before any material alteration in the weather felt a flight uneafinefs in the part where he received the blow; but when I met him lately, carrying a load on his fhoulders, he faid he was ftronger than before the accident, and that he now never experiences the leaft inconvenience in his back, either from working or the weather.

Various inftances have occurred of palfies, fomewhat fimilar to the prefent; but I have never heard, or read of one, where the difeafe fo foon arrived at fo great a height, where the cure was fo quickly completed, or where the remedy was more ftrongly marked with fuccefs. No one, I prefume, will hefitate to pronounce the cure to have been entirely effected by the iffue, as no other means were purfued, and every fymptom affumed a milder afpect as foon as it began to difcharge.

